# Impact of mask-wearing on emotion recognition accuracy and fixation duration in young children

**DOI:** 10.3389/fpsyg.2026.1788919

**Published:** 2026-03-24

**Authors:** Jeongeun Lee, Hyorim Lee, Minsol Kim, Chunghee Chung

**Affiliations:** 1 Busan Gender Equality and Family, Lifelong Education Institute, Busan, Republic of Korea; 2 Department of Home Economics Education, Kyungpook National University, Daegu, Republic of Korea; 3 Center for Beautiful Aging, Kyungpook National University, Daegu, Republic of Korea; 4 School of Child Studies, Kyungpook National University, Daegu, Republic of Korea

**Keywords:** childhood development, COVID-19, emotion cognition, eye movement, facial expressions, masks

## Abstract

**Introduction:**

Understanding how children recognize facial emotions is essential for explaining the development of social communication. During the COVID-19 pandemic, widespread mask-wearing partially occluded facial expressions, potentially influencing children's emotion recognition processes.

**Methods:**

This study investigated age-related differences in emotion recognition accuracy and gaze fixation patterns in young children using eye-tracking technology. Participants included 36 three-year-olds and 45 five-year-olds. Children viewed facial expressions representing four emotions (joy, sadness, anger, and fear) presented in both masked and unmasked conditions while fixation duration and recognition accuracy were recorded.

**Results:**

The results showed significant effects of age, emotion type, and mask-wearing on emotion recognition accuracy. Overall recognition accuracy decreased under masked conditions. Eye-tracking analysis revealed that children allocated significantly greater visual attention to the eye region when faces were masked, whereas gaze distribution between the eyes and mouth was more balanced in unmasked conditions.

**Discussion:**

These findings suggest that facial occlusion caused by mask-wearing alters visual attention strategies and emotion recognition processes in early childhood. The results provide important insights into how young children adapt their perceptual strategies when key facial cues are partially obscured.

## Introduction

During the COVID-19 pandemic, countries worldwide responded to the serious threat of mass infection with intensive measures such as traditional epidemiological investigations, regional lockdowns, activity restrictions, and travel bans. Health authorities in various countries also strongly encouraged their citizens to adopt preventive measures, including frequent hand washing, temperature checks, and improved ventilation. These efforts were complemented by policies promoting mask-wearing, particularly the consistent use of masks to ensure that both the nose and mouth were adequately covered. Regions like Hong Kong, Singapore, Japan, and South Korea adopted mask-wearing as a common practice early in the pandemic (Griffiths, 2020). In South Korea, for instance, mask-wearing was not merely recommended as a safety measure but was made mandatory for both children and adults.

Wearing masks is helpful in preventing infectious diseases; however, stringent measures, along with social distancing and self-quarantine, have led to changes in human social skills. Specifically, previous studies suggested that masks make facial recognition (Freud et al., 2020; Stajduhar et al., 2022) and emotional perception (Carbon and Serrano, 2021; Calbi et al., 2021) more difficult. Recent studies have further demonstrated that masks impair the recognition of basic facial emotions in both adults and children (Gori et al., 2024; Leitner et al., 2022; Mastorogianni et al., 2024).

Recent research has highlighted that the impact of facial masking on emotion processing is not uniform but depends critically on task relevance (Montalti and Mirabella, 2024). Specifically, surgical masks appear to disrupt behavioral and perceptual responses to emotional expressions primarily when emotional information is directly relevant to the observer's task goals. For instance, Montalti and Mirabella (2024) demonstrated that masks selectively impaired responses to happy expressions when participants were required to categorize facial emotions, whereas no such modulation emerged when emotional expressions were incidental to the task, such as during gender discrimination judgments. This task-relevance effect has been consistently supported in subsequent theoretical and empirical work (Maxwell et al., 2025; Mirabella and Montalti, 2025), suggesting that the social and perceptual consequences of facial occlusion are most pronounced in contexts where emotional cues guide interaction or decision-making.

In the present study, emotional information was explicitly task-relevant, as children were instructed to identify facial expressions. From this perspective, the observed masking effects on recognition accuracy and gaze allocation may reflect goal-directed perceptual adjustments rather than generalized perceptual disruption. Understanding how such task-dependent mechanisms operate in early childhood is particularly important, given that young children frequently rely on emotionally meaningful exchanges—especially with caregivers—to interpret social signals and regulate behavior.

In the initial 5 years of life, the ability to comprehend and accurately identify the emotions of others undergoes substantial refinement. Although infants can discriminate basic emotional expressions within the first year of life, reliable labeling and conceptual understanding of emotions develop gradually throughout early childhood. Emotion-related vocabulary typically emerges around age two, and by approximately 3 years of age, children begin to match verbal labels with prototypical facial expressions such as happy, sad, angry, and scared (Bretherton et al., 1986; Pons et al., 2004). Importantly, developmental progression differs across emotion types. Happiness is generally recognized first and with the highest accuracy, followed by sadness or anger, whereas fear remains the most challenging emotion for young children to identify (Camras and Allison, 1985; Pollak and Kistler, 2002). This hierarchical pattern suggests that emotion recognition abilities develop unevenly, with positive expressions preceding more complex or ambiguous negative emotions.

The primary goal is to gain insights into how these factors interact and impact emotion recognition skills among 3 to 5-year-olds, shedding light on the effects of mask-wearing and different emotion types on the developmental trajectory of facial expression recognition in this age group.

Research findings indicate a qualitative shift in the processing of masked faces (Freud et al., 2020). Conventional facial recognition is known to involve holistic processing, considering the face as a whole rather than individual parts. However, holistic processing decreases for masked faces (Stajduhar et al., 2022). In the context of emotion recognition from facial expressions, certain facial components, particularly the eyes and mouth, play significant roles. When portions of the face are occluded, as in mask-wearing contexts, children may rely more heavily on the remaining visible regions. Understanding how children allocate visual attention across facial regions is therefore critical for interpreting how masking may alter emotion processing.

Some scholars have employed eye-tracking to investigate facial recognition patterns (Arizpe et al., 2017; Royer et al., 2018). The eye-tracker device follows the eye movement of research participants in real-time to identify continuous changes in the recognition process (Holmqvist et al., 2011). Such tracking information enables researchers to pinpoint the area of interest (AOI) (Bolden et al., 2015) because the region where the gaze is fixated can be considered the focal area for cognitive processing to take place (Just and Carpenter, 1980).

While various studies have emphasized the significance of specific facial components, particularly the eyes and mouth, in the process of emotion recognition, their relative importance remains disputed. Some viewpoints propose that the significance of the eyes and mouth may vary based on the type of emotion being expressed (Hanawalt, 1944; Boucher and Ekman, 1975). Murphy and Isaacowitz (2010) observed that negative emotions were associated with a higher gaze fixation rate on the eye region, while positive emotions were linked to a higher gaze fixation rate on the mouth region. In contrast, other perspectives contend that either the eyes or the mouth can independently play a crucial role in emotion recognition, irrespective of the specific emotion being conveyed (Fridlund, 1994; de Bonis, 2004; Baron-Cohen et al., 1997).

In addition, previous research has used facial scanning to examine differences in AOIs across age groups (Haensel et al., 2020). Furthermore, research has demonstrated disparities in AOIs between adults and elderly individuals with superior emotion recognition accuracy (Royer et al., 2018). This study expands on previous research, anticipating that differences in the proportion of fixation duration on the eyes and mouth may occur based on mask-wearing, emotion type, and age.

This study aims to examine how mask-wearing, emotion type, and age collectively influence the ability of young children to accurately recognize facial expressions. Specifically, we investigate the differences in emotion recognition accuracy and proportion of fixation duration among children aged 3–5 years. This research is situated within the broader context of widespread mask use during the COVID-19 pandemic.

Based on these theoretical and empirical considerations, the present study addressed the following research questions:

1. Does the accuracy of emotion recognition from facial expressions differ depending on the children's age, the presence of masks, and the types of emotion?
2. Do children show different proportions of fixation durations on facial expression areas depending on the presence of masks and the types of emotion?

Although mask usage is not currently as prevalent, understanding these dynamics is crucial for informing educational and caregiving practices, especially given that masks were widely worn during the COVID-19 pandemic. The findings of this study provide valuable insights into the perceptual consequences of facial occlusion for emotion recognition processes in young children.

## Materials and methods

### Participants

The study participants comprised 3-year-old and 5-year-old children enrolled in kindergartens and daycare centers in Daegu Metropolitan City, South Korea. Informed consent forms were distributed to families, and written consent was obtained from legal guardians prior to participation.

A total of 100 children were initially recruited, including 48 three-year-olds and 52 five-year-olds. Of these, 19 children were excluded due to visual impairments (e.g., myopia or astigmatism), habitual use of corrective glasses, calibration failure, or not meeting predefined eye-tracking data quality criteria. The final analytic sample therefore consisted of 81 children, including 36 three-year-olds and 45 five-year-olds.

An *a priori* power analysis was conducted using G^*^Power 3.1 (Faul et al., 2007) to estimate the required sample size for detecting within–between interaction effects in a repeated-measures ANOVA design. Assuming a small effect size (*f* = 0.10), an alpha level of 0.05, and a desired statistical power of 0.80, the analysis indicated that a minimum total sample of 60 participants was required. The final analyzed sample of 81 children therefore exceeded this requirement. The mean age of the 3-year-old group (15 boys, 21 girls) was 50.4 months (range = 43–57, SD = 4.02). The mean age of the 5-year-old group (19 boys, 26 girls) was 74.8 months (range = 68–80, SD = 3.67).

**Figure 1:**
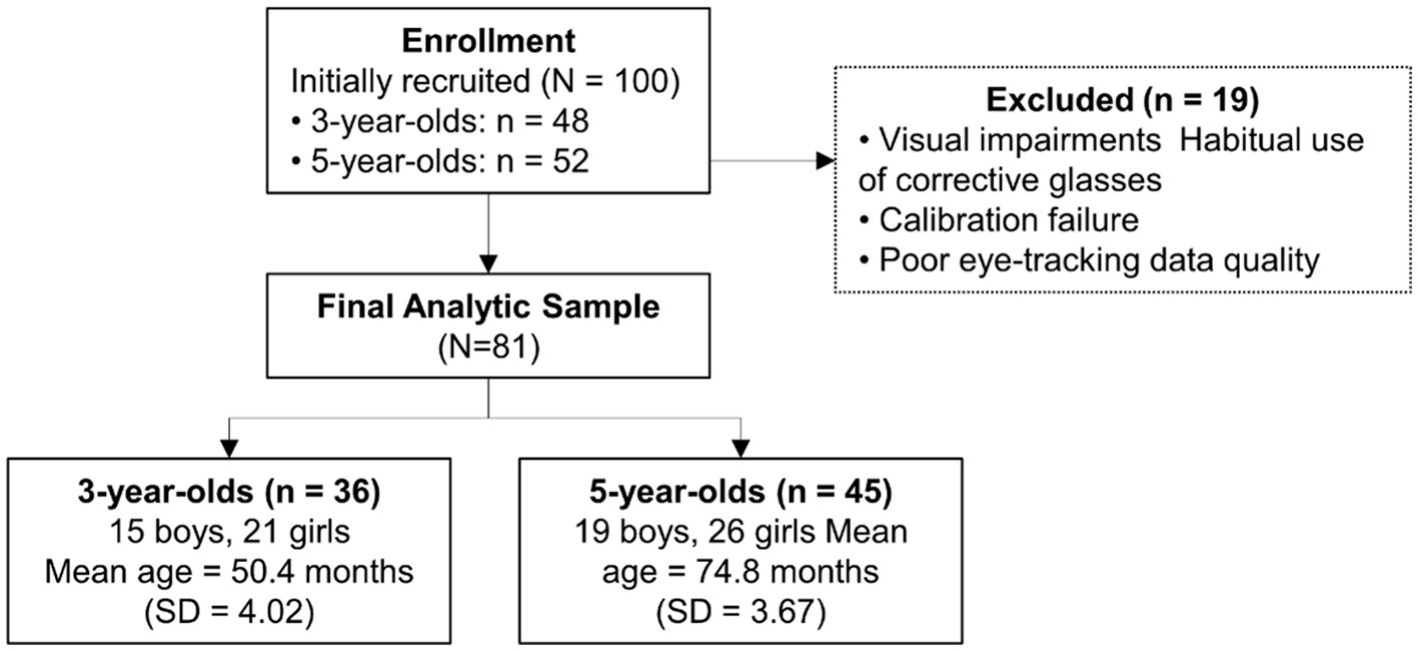
Participant recruitment and inclusion flow diagram. A total of 110 children were initially recruited (48 three-year-olds, 52 five-year-olds). 19 children were excluded due to visual impairments (e.g., myopia, astigmatism), habitual use of corrective glasses, calibration failure, or not meeting predefined eye-tracking data quality criteria. The final analytic sample consisted of 81 children (36 three-year-olds, 45 five-year-olds).

Within the Korean educational system, children typically enter kindergarten between ages 3 and 5, with the academic year running from March to February. Data collection was conducted between August 10 and October 18, corresponding to the middle of the academic year. A participant recruitment flow diagram is presented in Figure 1.

### Eye-tracking apparatus and geometric setup

Eye movements were recorded using a Tobii Pro X3-120 eye-tracker (Tobii AB, Stockholm, Sweden) (Tobii, 2022) mounted on a 24-inch monitor (53.1 cm wide × 29.9 cm high; resolution 1920 × 1080 pixels). Children were seated in front of the display with their chin placed on a chin rest to minimize head movements, and the experimenter ensured that the viewing distance was maintained at approximately 60–70 cm throughout the session.

A five-point calibration procedure was conducted prior to the experiment. Calibration was repeated once when necessary, and recordings were accepted when the validation error was below 1° of visual angle.

**Figure 2:**
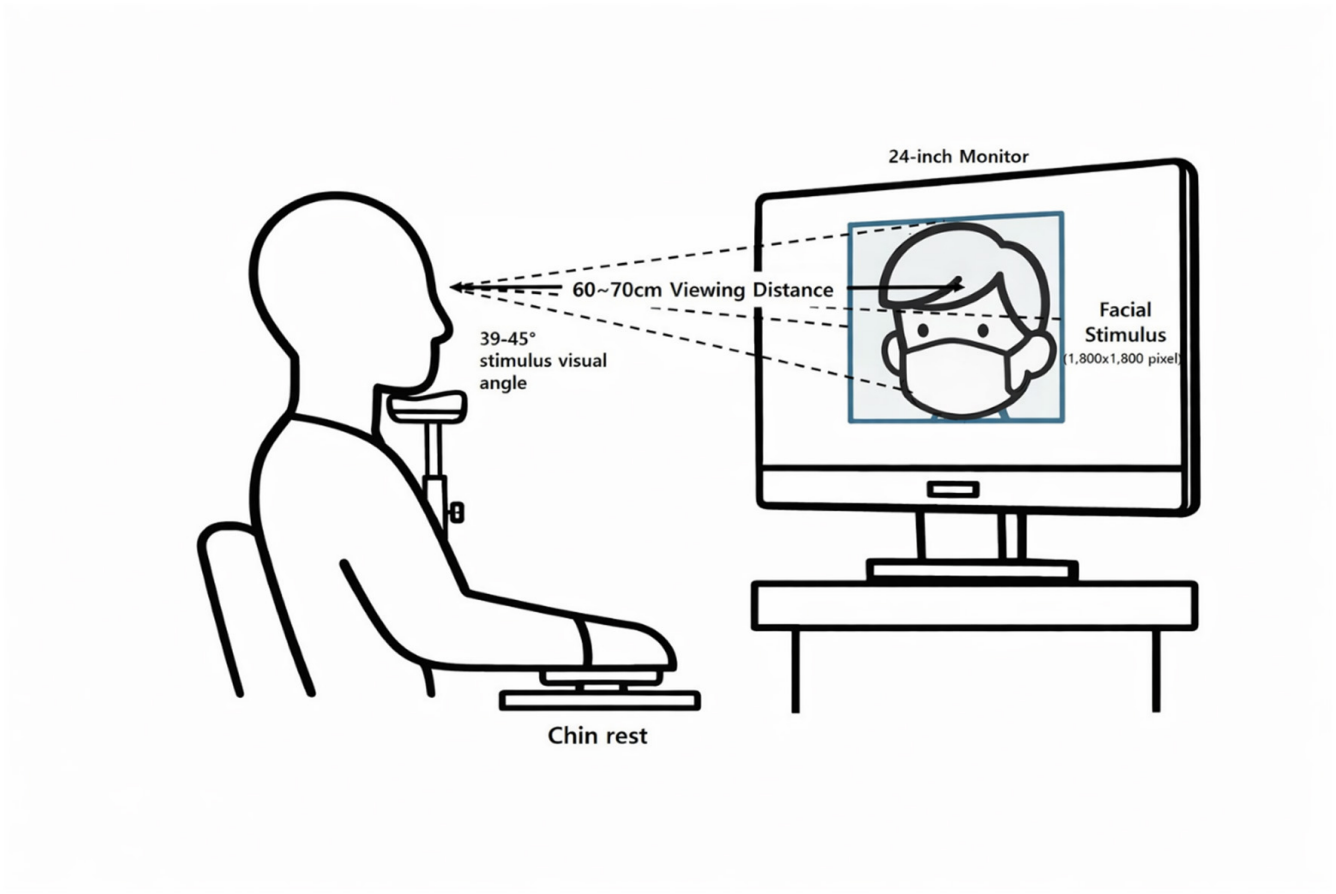
Experimental setup for the eye-tracking task. Children were seated approximately 60 cm from a 24-inch monitor while viewing facial stimuli. An eye-tracking device positioned below the monitor recorded gaze behavior. This schematic was Created using AI-assisted illustration tools.

Based on this geometric configuration, one degree of visual angle corresponded to approximately 42 pixels on the screen, such that a single pixel subtended about 0.02–0.03 degrees of visual angle. All facial stimuli were presented as 1,800 × 1,800 pixel square images centered on the screen, allowing the face region to occupy a substantial portion of the visual field. Gaze data were collected using Tobii Pro Lab (version 1.171) at a sampling rate of 120 Hz, which provides sufficient temporal resolution to capture fixations and saccades in young children. A schematic diagram of the experimental viewing arrangement is shown in Figure 2.

### Facial expression stimuli

Facial expression stimuli were derived from the Assessment of Children's Emotion Skills (ACES; Schultz et al., 2004), adapted for Korean children by Chung et al. (2022). Although the original ACES includes multiple sections (facial expressions, social situations, and social behaviors), only the facial expression section was used in the present study.

The original facial stimulus set consisted of 26 photographs representing four basic emotions (joy, sadness, anger, and fear), including blended expressions. For the purposes of experimental control and scoring feasibility, 16 photographs representing clearly identifiable emotional expressions were selected.

To examine the effect of mask-wearing on emotion recognition, surgical masks were digitally superimposed onto each facial photograph using Adobe Photoshop CC 2020, resulting in masked versions of all 16 stimuli. This produced a total of 32 stimulus trials (16 masked, 16 unmasked).

To ensure content validity, the masked stimuli were evaluated by three experts (one Ph.D. in early childhood education, one professor in child studies, and one professor in home economics education). Experts rated whether emotional expressions remained distinguishable despite partial facial occlusion using a 5-point Likert scale. All stimuli were deemed appropriate for use in the experimental task.

Each child viewed sixteen facial photographs presented in both masked and unmasked versions, resulting in a total of 32 stimulus trials.

### Calibration procedure

Before the main task, a five-point calibration procedure was conducted individually with each child to establish the mapping between eye position and gaze coordinates on the screen. Animated targets accompanied by sound effects were used to attract and sustain the children's attention as the calibration points appeared sequentially at different screen locations. Calibration was accepted when the average gaze error across the five points did not exceed 1.0 degree of visual angle, which was set as the criterion for adequate measurement accuracy in this age group. When the initial calibration failed to meet this criterion, the procedure was repeated; up to three calibration attempts were allowed for each participant. Children who did not achieve acceptable calibration after three attempts were excluded from participation. All children included in the final sample met the calibration accuracy criterion.

### Definition of areas of interest

**Figure 3:**
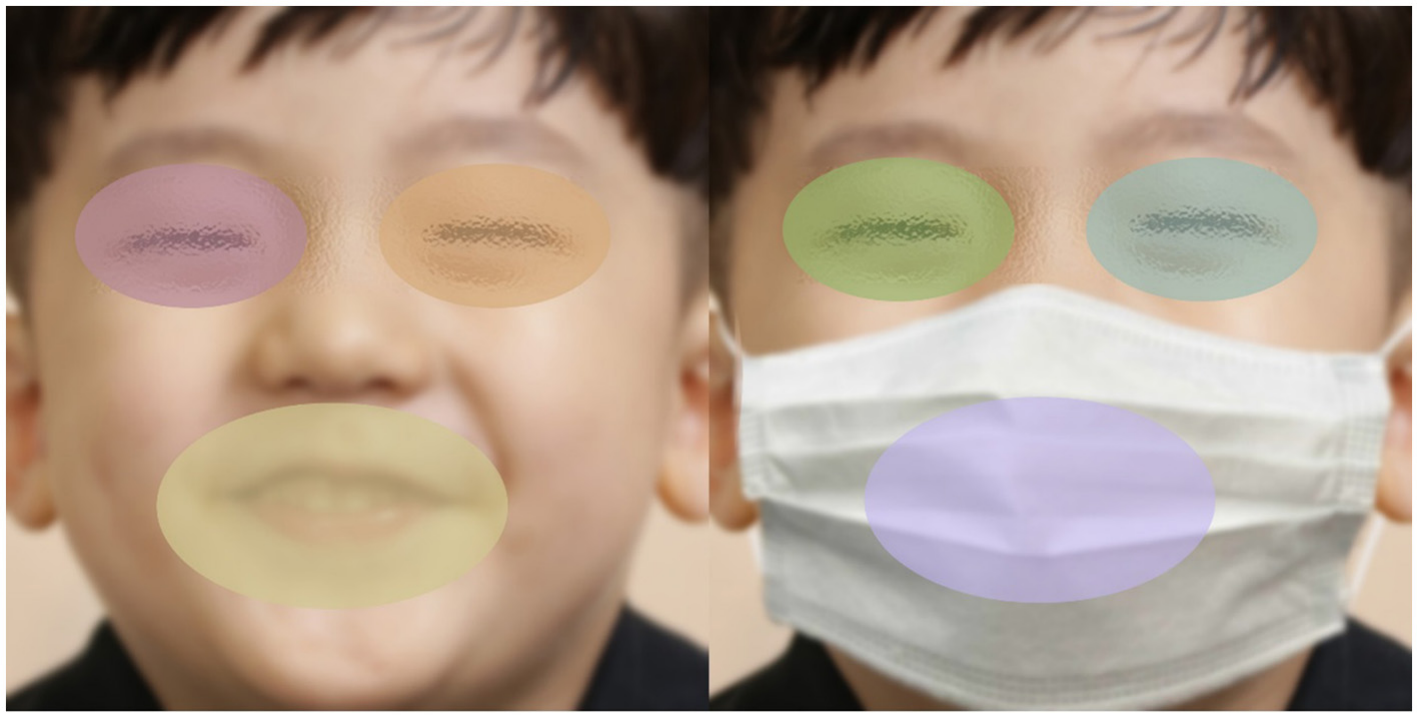
Example stimuli illustrating the areas of interest (AOIs). Colored overlays indicate the predefined eye and mouth regions for both unmasked and masked facial expressions.

To examine children's gaze allocation to diagnostically relevant facial regions, two elliptical areas of interest (AOIs) were defined on each stimulus: an eye AOI and a mouth AOI. The eye AOI encompassed both eyes and the surrounding periocular region and measured approximately 590 × 350 pixels, corresponding to about 14.1° × 8.4° of visual angle. The mouth AOI covered the mouth and perioral area and measured approximately 880 × 470 pixels, corresponding to about 21.1° × 11.3° of visual angle. The vertical distance between the centroids of the eye and mouth AOIs was approximately 240–260 pixels, which corresponds to about 5.8–6.2 degrees of visual angle. This spatial separation is substantially larger than the reported accuracy (0.4°) and precision (0.24°) of the Tobii Pro X3-120, indicating that the eye-tracker can reliably discriminate between gaze directed to the eyes vs. the mouth. Because the spatial structure of the face remained identical across masked and unmasked versions, the same AOI definitions were applied across both conditions. A schematic illustration of AOI placement is provided in Figure 3. AOIs were defined and applied using Tobii Pro Lab software.

### Event detection and data processing

Raw gaze samples were processed in Tobii Pro Lab using the default I-VT (Identification by Velocity Threshold) fixation classifier. Gaze samples were classified as belonging to a fixation when the eye movement velocity was at or below 30 degrees per second, which is the standard velocity threshold in the I-VT algorithm. A minimum fixation duration of 60 ms was required; gaze events shorter than this duration were discarded to reduce the influence of noise and micro-movements that are unlikely to reflect meaningful visual processing. Velocity estimates were computed using a 20 ms time window, and a three-sample moving median filter was applied to the gaze signal to attenuate high-frequency noise. Adjacent fixations were merged into a single fixation when the temporal gap between them was 75 ms or less and the angular difference between their centroids was 0.5 degrees of visual angle or less, thereby preventing brief interruptions in tracking from artificially splitting a single fixation into multiple events. For all analyses, binocular gaze data were processed using the average position of the two eyes.

### Treatment of missing data and data quality

During data collection, gaps in the gaze signal occurred due to blinks, transient occlusion by eyelashes, or instances in which children briefly looked away from the screen. In the present study, gap-fill interpolation was disabled such that missing data segments were not artificially reconstructed; instead, these segments were treated as missing and excluded from fixation classification and AOI-based analyses. As a result, fixation, dwell time, and AOI hit measures were computed only from periods with valid, continuously recorded gaze data, adopting a conservative approach that avoids overestimating visual attention during intervals when children were not actually viewing the stimuli. At the trial level, trials with insufficient tracking (e.g., very low tracking ratio or no fixations within the predefined AOIs) were excluded from the analysis; participant- and trial-level inclusion criteria were applied to ensure that the reported fixation measures are based on gaze data of acceptable quality.

### Computation of fixation measures

For each trial, fixation duration within each AOI was defined as the cumulative time during which the child's gaze was classified as a fixation inside that AOI. Total fixation duration for a given trial or condition was obtained by summing the durations of all fixations across the eye and mouth AOIs. To index the relative allocation of visual attention, the proportion of fixation to each AOI was calculated by dividing the AOI-specific total fixation duration by the overall total fixation duration and multiplying by 100, yielding a percentage score. Thus, a proportion of 40% for the eye AOI indicates that 40% of the child's total fixation time during the trial was spent looking at the eye region. All fixation durations are reported in seconds, and all proportion-of-fixation measures are reported as percentages.

### Experimental procedure

Prior to testing, brief rapport-building interactions were conducted to familiarize children with the experimental setting. Children were provided with simple verbal instructions and examples to ensure comprehension of the four target emotions. Subsequently, the experiment was introduced to the participating children, who were instructed to sit upright in the chair with their chin on the chin rest to ensure that their gaze was accurately positioned for optimal eye-tracking calibration. Calibration was then conducted following the procedure described above.

Prior to the main experiment, a preliminary experiment was conducted on August 8–9, 2022, to refine the procedures and ensure the effectiveness of the experimental setup. Following this, the main experiment was carried out between August 10 and October 18, 2022.

Stimulus trials were presented across two sessions scheduled at least 1 day apart to reduce fatigue. The entire experimental procedure required approximately 15 min per child.

### Analysis

In this study, children's responses were collected to investigate potential variations in recognition accuracy and eye movement patterns concerning age, mask-wearing, and types of observed emotions in facial expressions. All statistical analyses were conducted using jamovi 2.3.18 (The jamovi project, Sydney, Australia). First, descriptive statistics (mean, standard deviation, skewness, kurtosis) were calculated for the accuracy of emotion recognition and fixation duration on AOI regions to check the assumptions of normal distribution. Next, a mixed repeated-measure analysis of variance (ANOVA) was employed, with age (3-year-old group, 5-year-old group) as a between-subject factor and emotion types (joy, sadness, anger, fear) and mask-wearing (not wearing, wearing) as within-subject factors. This analysis aimed to identify differences in facial expression recognition accuracy based on age, emotion type, and mask-wearing. Subsequently, another mixed repeated-measure ANOVA was carried out with age as a between-subject factor and emotion type, mask-wearing, and AOIs as within-subject factors. This analysis was conducted to investigate variations in eye movement patterns based on age, emotion type, and mask-wearing. Effect sizes (partial η^2^) were reported for all significant effects. *Post-hoc* comparisons were conducted using Bonferroni-adjusted pairwise tests where appropriate. For *post-hoc* comparisons, effect sizes (Cohen's *d*) were additionally reported.

## Results

### Accuracy in recognizing facial expressions

An analysis of the accuracy in recognizing facial expressions was conducted using a mixed repeated-measures ANOVA with Type III sums of squares. The analysis examined variations in emotion recognition accuracy as a function of age (3-year-olds vs. 5-year-olds), emotion type (joy, sadness, anger, fear), and mask-wearing condition (unmasked vs. masked). Descriptive statistics are presented in Supplementary Table S1. All variables met normality assumptions, with skewness values ≤ 3 and kurtosis values ≤ 8 (Kline, 2010).

Mauchly's tests of sphericity indicated violations for emotion type (*W* = 0.82, *p* = 0.010) and the emotion × mask interaction (*W* = 0.85, *p* = 0.031); therefore, Greenhouse–Geisser corrections were applied.

The main effect of age was statistically significant, *F*_(1, 79)_ = 13.44, *p* < 0.001, $\eta_{p}^{2}$ = 0.145. Significant main effects were also observed for emotion, *F*_(2.69, 212.28)_ = 12.75, *p* < 0.001, $\eta_{p}^{2}=$ 0.139, and mask condition, *F*_(1, 79)_ = 22.19, *p* < 0.001, $\eta_{p}^{2}=$ 0.219. The three-way interaction among emotion type, mask-wearing, and age was not statistically significant, *F*_(2.70, 213.01)_ = 1.31, *p* = 0.274, $\eta_{p}^{2}=$ 0.016.

However, a significant interaction was observed between emotion type and mask-wearing, *F*_(2.81, 222.20)_ = 2.83, *p* = 0.043, $\eta_{p}^{2}=$ 0.035. Bonferroni-adjusted *post-hoc* comparisons were conducted, and effect sizes (Cohen's *d*) were additionally reported.

**Figure 4:**
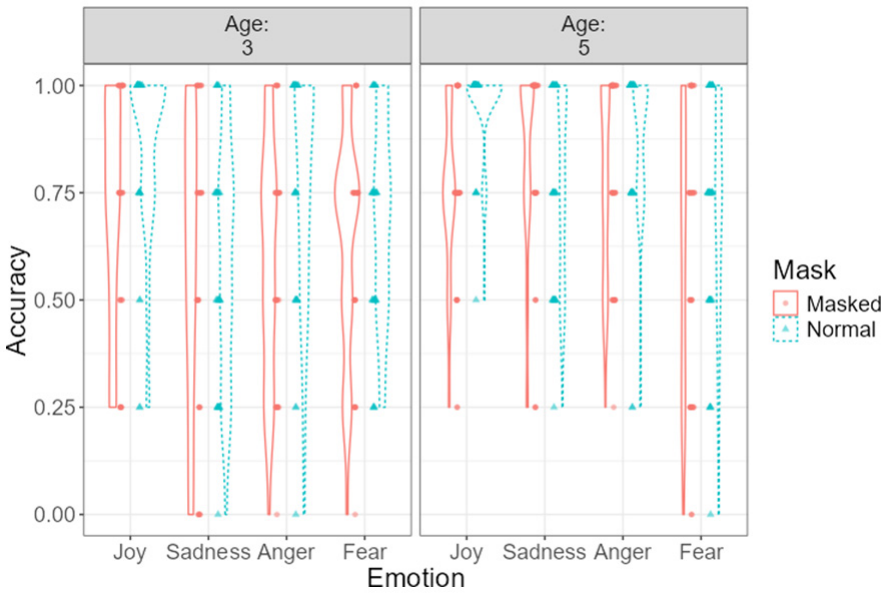
Emotion recognition accuracy as a function of mask condition and emotion type, displayed separately for 3- and 5-year-olds. Violin plots illustrate the distribution of individual accuracy scores across conditions. Dots represent individual participants, and error bars indicate ±1 standard error.

As illustrated in Figure 4, recognition accuracy declined under masked conditions across both age groups. However, the three-way interaction was not statistically significant; therefore, any apparent age-related differences in the magnitude of decline should be interpreted descriptively rather than inferentially.

### The interaction effects between age and emotion type

**Figure 5:**
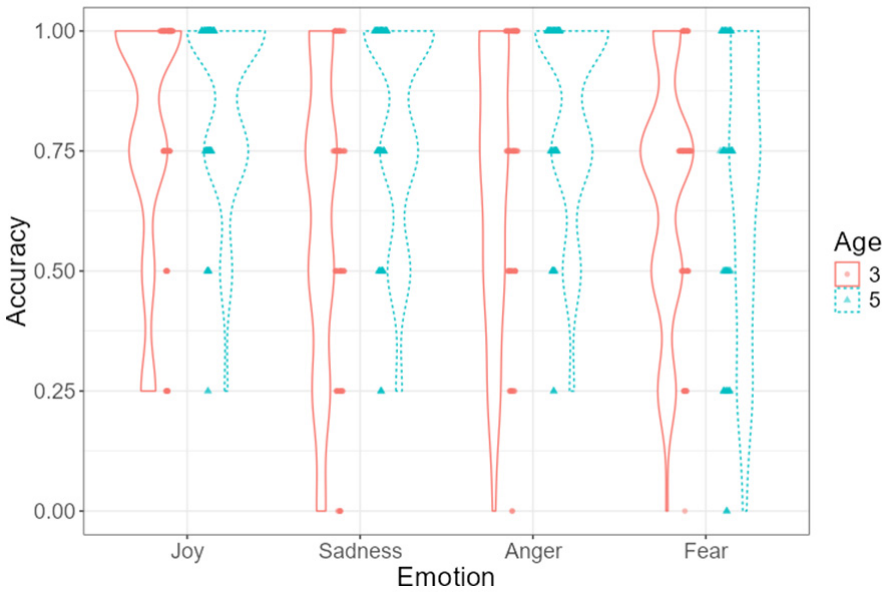
Emotion recognition accuracy across emotion types for 3- and 5-year-olds. Violin plots display the distribution of individual accuracy scores. Dots represent individual observations, and error bars denote ±1 standard error.

Figure 5 illustrates the interaction between age group and emotion type on emotion recognition accuracy. Estimated marginal means indicated that recognition accuracy differed across emotions and age groups, with generally higher accuracy observed among 5-year-olds compared to 3-year-olds.

Across both age groups, joy was recognized with the highest accuracy, whereas fear showed the lowest recognition performance. Age-related differences were particularly pronounced for sadness and anger, for which 5-year-olds demonstrated substantially higher recognition accuracy than 3-year-olds. This pattern indicates comparatively lower recognition accuracy for fear relative to other emotions. Bonferroni-adjusted *post-hoc* comparisons indicated that 3-year-olds recognized joy significantly more accurately than sadness, *t*_(79)_ = 5.15, *p* < 0.001, and fear, *t*_(79)_ = 3.31, *p* = 0.039. Among 5-year-olds, recognition accuracy for joy, sadness, and anger was significantly higher than for fear (all *p*s < 0.05). In addition, 5-year-olds recognized sadness, *t*_(79)_ = −4.31, *p* = 0.001, and anger, *t*_(79)_ = −3.30, *p* = 0.040, more accurately than 3-year-olds. Effect sizes (Cohen's *d*) for all pairwise comparisons are reported in Supplementary Table S1.

### The interaction effects between emotion type and mask-wearing

**Figure 6:**
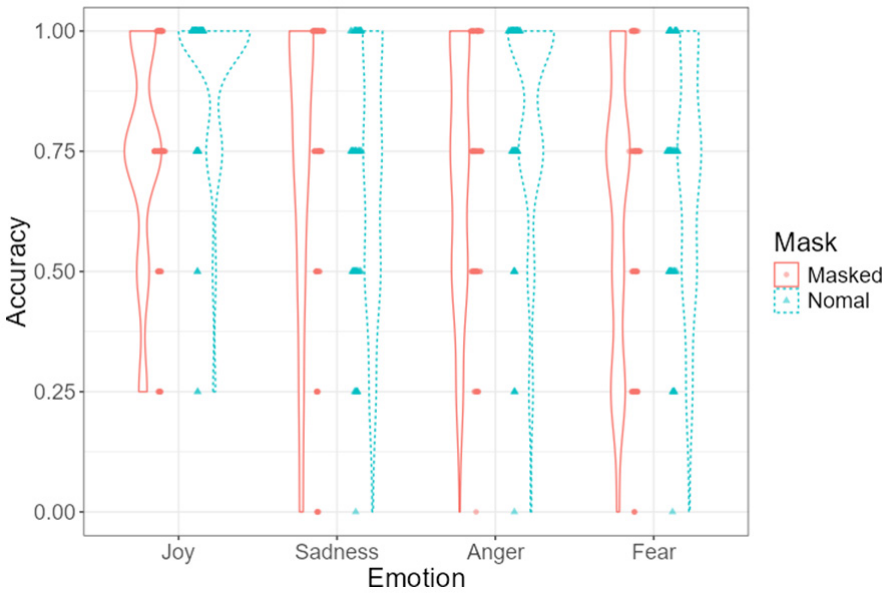
Distribution of recognition accuracy across emotion types by mask condition. Violin plots display individual data points with superimposed mean values and standard error bars.

Figure 6 illustrates the interaction between emotion type and mask-wearing on children's facial emotion recognition accuracy. Estimated marginal means indicated differential effects of mask-wearing across emotion categories.

Estimated marginal means indicated larger accuracy differences between masked and unmasked conditions for joy and anger than for sadness and fear.

*Post-hoc* Bonferroni-adjusted comparisons revealed that children recognized joy [*t*_(79)_ = 6.91, *p*_adj < 0.001] and anger [*t*_(79)_ = 3.32, *p*_adj = 0.038] significantly more accurately in unmasked faces than in masked faces.

No significant differences were observed for sadness [*t*_(79)_ = −1.03, *p*_adj = 0.311] or fear [*t*_(79)_ = 2.14, *p*_adj = 0.072].

Further comparisons within the unmasked condition indicated that recognition accuracy for joy was significantly higher than for sadness [*t*_(79)_ = 7.28, *p*_adj < 0.001], anger [*t*_(79)_ = 3.42, *p*_adj = 0.028], and fear [*t*_(79)_ = 6.96, *p*_adj < 0.001]. Additionally, anger was recognized more accurately than sadness [*t*_(79)_ = −4.39, *p*_adj < 0.001] and fear [*t*_(79)_ = 3.54, *p*_adj = 0.019].

No significant differences in recognition accuracy across emotion types were observed in the masked condition.

### Proportion of fixation duration in recognizing facial expressions

An analysis of fixation duration proportions was conducted using a mixed repeated-measures ANOVA with Type III sums of squares to examine variations in gaze allocation as a function of age (3 vs. 5 years), emotion type (joy, sadness, anger, fear), mask-wearing (unmasked vs. masked), and areas of interest (AOIs: eyes vs. mouth). Descriptive statistics for fixation duration proportions are presented in Supplementary Table S2. All variables satisfied normality assumptions, with skewness values ≤ 3 and kurtosis values ≤ 8 (Kline, 2010).

Mauchly's tests of sphericity indicated violations for emotion type (*W* = 0.87, *p* = 0.048) and the emotion × mask × AOI interaction (*W* = 0.85, *p* = 0.030). Accordingly, Greenhouse–Geisser corrections were applied to these effects.

The repeated-measures ANOVA revealed no significant main effect of age, *F*_(1, 79)_ = 0.05, *p* = 0.822, nor a significant four-way interaction among emotion type, mask-wearing, AOIs, and age, *F*_(2.71, 214.09)_ = 0.45, *p* = 0.699.

However, significant main effects were observed for emotion type, *F*_(2.73, 215.55)_ = 15.02, *p* < 0.001, $\eta_{p}^{2}=$ 0.160; mask-wearing, *F*_(1, 79)_ = 42.56, *p* < 0.001, $\eta_{p}^{2}=$ 0.350; and AOIs, *F*_(1, 79)_ = 280.72, *p* < 0.001, $\eta_{p}^{2}=$ 0.780.

Importantly, a significant three-way interaction emerged among emotion type, mask-wearing, and AOIs, *F*_(2.71, 214.20)_ = 40.70, *p* < 0.001, $\eta_{p}^{2}=$ 0.340. Bonferroni-adjusted *post-hoc* comparisons were subsequently conducted to further examine this interaction.

### The interaction effects among emotion type, mask-wearing, and AOIs

**Figure 7:**
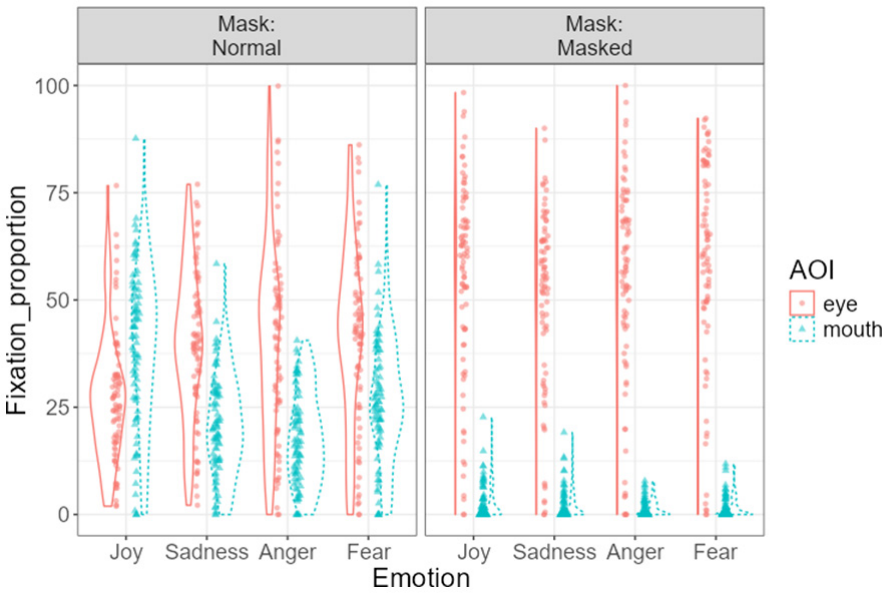
Three-way interaction among emotion type, mask condition, and area of interest (AOI) on fixation duration proportion. Separate panels display gaze allocation under normal and masked conditions. Violin plots illustrate the distribution of fixation proportions across conditions. Dots represent individual observations, and error bars indicate ±1 standard error.

Figure 7 illustrates the three-way interaction among emotion type, mask-wearing, and areas of interest (AOIs) on the proportion of fixation duration during facial expression processing. Estimated marginal means indicated distinct gaze allocation patterns depending on masking condition and emotional category.

For the eye AOI, fixation proportions increased substantially under masked conditions across all emotions. Specifically, for joyful expressions, eye fixation increased from 27.68% [SE = 1.65, 95% CI (24.39, 30.97)] in the unmasked condition to 55.16% [SE = 2.60, 95% CI (49.98, 60.34)] in the masked condition. A similar pattern was observed for sadness [unmasked: 39.54%, SE = 1.93, 95% CI (35.70, 43.38); masked: 49.58%, SE = 2.46, 95% CI (44.68, 54.47)], anger [unmasked: 40.92%, SE = 2.49, 95% CI (35.96, 45.87); masked: 54.15%, SE = 2.74, 95% CI (48.69, 59.60)], and fear [unmasked: 41.65%, SE = 2.29, 95% CI (37.09, 46.20); masked: 58.56%, SE = 2.76, 95% CI (53.06, 64.06)].

In contrast, fixation to the mouth AOI decreased dramatically when faces were masked. For joy, mouth fixation dropped from 39.00% [SE = 2.06, 95% CI (34.90, 43.09)] in the unmasked condition to 2.61% [SE = 0.46, 95% CI (1.69, 3.52)] in the masked condition. Comparable reductions were observed for sadness (20.62% → 2.07%), anger (16.99% → 1.21%), and fear (26.62% → 1.54%), all indicating substantial reductions in mouth-directed gaze when masks were present. Across most emotional expressions, children fixated significantly more on the eyes than on the mouth when processing masked faces. In contrast, in the unmasked condition joyful expressions elicited comparable fixation to the mouth and eyes, whereas negative emotions (sadness, anger, fear) were associated with relatively greater eye fixation.

Pairwise comparisons examining differential fixation between the eye and mouth regions across emotion types and masking conditions are presented in Table 1.

**Table 1:** Differential eye–mouth fixation proportion across emotion types and mask-wearing conditions.

**Eye–mouth**	**Mean difference**	**SE**	**df**	***T***	***p*_adj**
**Joy**					
Without mask	−11.32	3.11	79	−3.642	0.058
With mask	52.55	2.74	79	19.184	<0.001
**Sadness**					
Without mask	18.91	2.62	79	7.222	<0.001
With mask	47.51	2.51	79	18.914	<0.001
**Anger**					
Without mask	23.92	2.64	79	9.077	<0.001
With mask	52.94	2.77	79	19.123	<0.001
**Fear**					
Without mask	15.03	2.96	79	5.082	<0.001
With mask	57.02	2.81	79	20.324	<0.001

In the unmasked condition, fixation allocation varied depending on emotion type. For joyful expressions, fixation to the mouth did not differ significantly from fixation to the eyes after Bonferroni correction (Mean difference = −11.32, *p* = 0.058). In contrast, for negative emotions—including sadness, anger, and fear—children fixated significantly more on the eyes than on the mouth (all *p*s < 0.001).

In the masked condition, fixation to the eyes was significantly greater than to the mouth across all emotion types. Eye–mouth differences were particularly pronounced for fear (Mean difference = 57.02), anger (52.94), and joy (52.55), all *p*s < 0.001. These findings indicate a robust shift toward eye-dominant gaze processing when lower facial information was occluded.

Direct comparisons between masked and unmasked conditions for each AOI are summarized in Table 2.

**Table 2:** Contrasting eye–mouth fixation proportion in masked vs. unmasked faces.

**Masked–unmasked faces**	**Mean difference**	**SE**	**df**	***T***	***p*_bonferroni_**
**Joy**					
Eye	−27.48	2.48	79	−11.087	<0.001
Mouth	36.39	2.06	79	17.705	<0.001
**Sadness**					
Eye	−10.04	1.93	79	−5.199	<0.001
Mouth	18.55	1.23	79	15.145	<0.001
**Anger**					
Eye	−13.23	2.03	79	−6.523	<0.001
Mouth	15.78	1.13	79	13.957	<0.001
**Fear**					
Eye	−16.91	2.22	79	−7.631	<0.001
Mouth	25.08	1.63	79	15.438	<0.001

Across all emotion types, eye fixation proportions were significantly higher in the masked condition than in the unmasked condition (all *p*s < 0.001). Conversely, mouth fixation proportions were significantly higher in the unmasked condition than in the masked condition (all *p*s < 0.001). These comparisons are summarized in Table 3.

**Table 3:** Effect of emotion, AOIs, and masks on fixation duration.

**Mask-wearing**	**AOIs**	**Emotion type**		**Mean difference**	**SE**	**df**	***T***	***p*_bonferroni_**
Without mask	Eye	Joy	–Sadness	−11.86	1.77	79	−6.696	<0.001
		Joy	–Anger	−13.24	2.44	79	−5.437	<0.001
		Joy	–Fear	−13.97	1.96	79	−7.131	<0.001
		Sadness	–Anger	−1.38	2.27	79	−0.608	1
		Sadness	–Fear	−2.11	2.03	79	−1.038	1
		Anger	–Fear	−0.73	1.87	79	−0.39	1
	Mouth	Joy	–Anger	22.00	1.73	79	12.699	<0.001
		Joy	–Fear	12.38	1.84	79	6.724	<0.001
		Joy	–Sadness	18.37	2.04	79	9.012	<0.001
		Sadness	–Anger	3.63	1.39	79	2.622	1
		Sadness	–Fear	−5.99	1.50	79	−4.004	0.017
		Anger	–Fear	−9.63	1.42	79	−6.777	<0.001
With mask	Eye	Joy	–Sadness	5.59	2.00	79	2.8	0.77
		Joy	–Anger	1.02	1.77	79	0.574	1
		Joy	–Fear	−3.40	1.94	79	−1.753	1
		Sadness	–Anger	−4.57	1.88	79	−2.433	1
		Sadness	–Fear	−8.98	1.80	79	−5.001	<0.001
		Anger	–Fear	−4.41	1.82	79	−2.426	1
	Mouth	Joy	–Sadness	0.54	0.59	79	0.911	1
		Joy	–Anger	1.40	0.51	79	2.721	0.961
		Joy	–Fear	1.07	0.54	79	1.98	1
		Sadness	–Anger	0.86	0.47	79	1.825	1
		Sadness	–Fear	0.53	0.46	79	1.165	1
		Anger	–Fear	−0.33	0.34	79	−0.96	1

This pattern indicates that occlusion of the lower face was associated with increased eye fixation and reduced mouth fixation.

## Discussion

### Accuracy in recognizing facial expressions

This study investigated the accuracy of emotion recognition in facial expressions among 3- and 5-year-olds, revealing significant main effects of mask-wearing, age, and emotion type. Overall, young children exhibited higher accuracy in recognizing emotions in unmasked faces than in masked faces, and 5-year-olds demonstrated higher accuracy than 3-year-olds. Recognition accuracy also varied by emotion type. However, significant interactions between emotion type and age, as well as between emotion type and mask-wearing, were also observed, indicating that the influence of mask-wearing on emotion recognition varied depending on specific emotional categories. Similar patterns have been reported in adults, where face masks generally reduce the recognition of basic emotions and particularly impair the identification of happiness compared to some negative emotions such as sadness or fear (Leitner et al., 2022).

The present findings can also be interpreted within the task-relevance framework of emotion processing. Recent research suggests that the disruptive effects of face masks on emotional perception are not uniform but depend on whether emotional information is directly relevant to the observer's task goals (Montalti and Mirabella, 2024; Maxwell et al., 2025; Mirabella and Montalti, 2025).

In addition to task relevance, emotional arousal has been identified as another critical factor modulating the impact of facial occlusion on emotion perception. Prior adult studies have demonstrated that mask-related impairments in emotion recognition vary as a function of stimulus arousal level, with highly arousing expressions (e.g., fear or anger) sometimes remaining relatively more detectable despite partial facial obstruction, whereas low-arousal expressions show greater vulnerability to occlusion effects. These findings suggest that perceptual reliance on diagnostic facial cues may shift depending not only on task demands but also on the emotional intensity conveyed by the stimulus.

In experimental contexts requiring explicit emotion identification, masks appear to interfere more strongly with behavioral responses because facial cues necessary for accurate emotion identification become central to task performance. By contrast, when emotional expressions are incidental or task-irrelevant, mask-related effects tend to be attenuated or absent. Because children in the present study were explicitly instructed to identify emotional expressions, the observed reductions in recognition accuracy under masked conditions are consistent with this task-relevance account. From this perspective, the findings suggest that mask-related perceptual constraints may become more apparent in situations where children are required to actively interpret emotional cues, such as during emotionally meaningful interactions. At the same time, this framework suggests that mask effects are context-dependent rather than universal.

Children's ability to recognize emotions from facial expressions varied by age and emotion type. No significant differences in the assessment of joy and fear were observed between the two age groups, indicating comparable recognition levels. In contrast, a marked divergence emerged in the recognition of sadness and anger between the two groups. Specifically, 5-year-olds demonstrated significantly higher accuracy in discerning sadness and anger compared to their 3-year-old counterparts. Additionally, both age groups recognized the emotion of joy with high accuracy and no significant age-based differences.

This finding aligns with previous research suggesting that children are sensitive to the emotion of joy from an early age (Dewitte and De Houwer, 2008; Horstmann, 2003; Reed et al., 2018). This result is also consistent with the notion that the ability to recognize positive emotions develops before the capacity to discern negative emotions (Boyatzis et al., 1993; Camras and Allison, 1985; Sroufe, 1997; Widen and Russell, 2003). Additionally, it corroborates previous studies indicating that joy is one of the most accurately identified emotions in facial expressions (Calder et al., 2000; Calvo and Lundqvist, 2008; Palermo and Coltheart, 2004; Tottenham et al., 2009). Therefore, the lack of a significant age-group difference in recognizing joy may reflect a ceiling effect (Vicari et al., 2000).

In contrast, 3- and 5-year-olds recognized fear in facial expressions with an accuracy of 67%, which was less accurate than the identification of other emotions. The absence of significant age-group differences in recognizing fear may appear counterintuitive, despite prior reports that caregivers often label fear-related situations in early childhood (Camras and Sachs, 1991; Rosen et al., 1992). Alternatively, these results might stem from children's relatively lower sensitivity to fear, as they may not frequently encounter this emotion in their daily lives (Gao and Maurer, 2009; Malatesta and Haviland, 1982).

A notable difference emerged between the two age groups regarding the recognition accuracy of sadness and anger. Three-year-olds recognized joy more accurately than sadness and fear, while 5-year-olds recognized joy, sadness, and anger more accurately than fear. These findings suggest that emotion recognition skills, particularly for the emotions of sadness and anger, develop significantly during early childhood (Griffiths, 2020; Stajduhar et al., 2022; Carbon and Serrano, 2021), as evidenced by the children participating in this study. Longitudinal findings further indicate that toddlers exposed to masked faces during the COVID-19 pandemic can improve recognition of masked expressions over time (Gori et al., 2024). However, the present cross-sectional design does not allow conclusions regarding adaptive developmental change in response to sustained mask exposure.

In the *post-hoc* analysis examining the interaction effects of mask-wearing and emotion type on the accuracy of facial expression recognition, the results indicated that when observing unmasked faces, joy was recognized most accurately, followed by anger and then sadness and fear, with differences in recognition accuracy based on emotion type. However, when it came to recognizing emotions in masked faces, no significant variations were observed based on emotion type. Moreover, differences in recognition accuracy based on mask-wearing were evident only for joy and anger.

Participants showed reduced recognition accuracy for joy—and to a lesser extent anger—when observing masked faces. This finding aligns with previous research, which suggests that accurate and rapid recognition of joy occurs when the entire face or mouth area is visible, and recognition accuracy significantly diminishes when only the eye area is presented (Fridlund, 1994; Baron-Cohen et al., 2001; Haensel et al., 2020). This phenomenon may be attributed to the unique mouth shape associated with expressions of joy, a characteristic that distinguishes it from other emotions (Calvo and Marrero, 2009; Kohler et al., 2004). Additionally, this observation is consistent with prior reports indicating that mask-wearing hinders the accurate detection of positive emotions more than the correct identification of negative emotions (Fischer et al., 2012). Recent work with school-age children similarly shows that emotion recognition from masked faces is reduced relative to unmasked faces and can be further modulated by children's current affective state (Mastorogianni et al., 2024).

In the context of recognizing negative emotions, this study found differences in recognition accuracy for anger. According to previous research, Korean children tend to focus on the nose when recognizing anger in facial expressions (Chung et al., 2023), which could explain the difficulties they experience in emotion recognition when a mask conceals this area. However, while the negative effects of mask-wearing on the expression of joy are relatively consistent, the impact of mask-wearing on recognizing negative emotions in facial expressions varies across studies (de Bonis, 2004; Royer et al., 2018; Pazhoohi et al., 2021; Ruba and Repacholi, 2020). Therefore, further investigations involving diverse participant groups are warranted to more comprehensively understand the effects of mask-wearing on the recognition of negative emotions in facial expressions.

### Eye-tracking results

Repeated-measures ANOVAs were conducted to investigate potential differences in the proportion of fixation duration between 3-year-old and 5-year-old children during their facial expression recognition processes. These analyses factored in the variables of mask-wearing, emotion type, age, and AOIs, specifically focusing on the eyes and mouth. The results indicated that the primary effect of age was not statistically significant, suggesting that overall gaze allocation patterns were comparable between 3- and 5-year-olds when recognizing emotions. However, significant interactions were observed among mask-wearing, emotion type, and AOIs, indicating variations in the proportion of fixation duration for each AOI region based on mask-wearing and the specific emotion type.

When examining unmasked faces expressing joy, the proportion of fixation durations for the eyes and mouth did not exhibit significant differences. In contrast to joyful expressions, children showed higher fixation proportions on the eyes than on the mouth for sadness, anger, and fear across both mask conditions. In addition, mouth-directed fixation was relatively greater for joy than for sadness, anger, or fear. These findings are consistent with prior research, underscoring the pivotal role of the eyes in processing visual information for facial expressions, particularly concerning emotions like sadness, anger, and fear (Murphy and Isaacowitz, 2010; Bassili, 1979; Peterson and Eckstein, 2012; Baron-Cohen et al., 2001; Lewis and Edmonds, 2003; Smith et al., 2005).

Additionally, these results align with previous studies, which have also shown that negative emotions are associated with a higher gaze fixation rate on the eye region, whereas positive emotions are linked to a higher gaze fixation rate on the mouth region (Murphy and Isaacowitz, 2010; Wegrzyn et al., 2017). Regarding joy, the extended gaze duration on the mouth is attributed to the distinct “U” shape it forms, setting it apart from other emotions characterized by differing mouth shapes (Pazhoohi et al., 2021; Ekman and Friesen, 1976; Calvo and Nummenmaa, 2008).

Furthermore, when comparing gaze durations between masked and unmasked faces, the proportions of participants' fixation duration on the eyes and mouth increased and decreased, respectively, when observing masked faces. The observed redistribution of gaze toward the eye region under masked conditions likely reflects a compensatory perceptual strategy in response to reduced availability of lower facial cues. Within a task-relevance framework, these perceptual adjustments may become particularly evident when emotional interpretation is explicitly required, as in the present emotion-identification task. For instance, studies conducted in the U.S. have shown that observers increase reliance on eye-region cues when interacting with masked faces (Barrick et al., 2021).

When recognizing fear in masked faces, gaze durations on the eyes were significantly longer than when recognizing sadness. The identification of sadness and fear primarily relies on eye cues (Wegrzyn et al., 2017). Considering the earlier results regarding emotion recognition accuracy, both sadness and fear are challenging emotions to recognize. However, the recognition of sadness appears to be a developmental task, particularly between the ages of three and five. In contrast, recognizing fear remains a challenging task, even for 5-year-old children.

Individuals who are less experienced in information processing tend to exhibit longer fixation durations than those with more experience (Brams et al., 2019). Therefore, when children rely on limited cues, such as the eyes, to interpret emotions in the context of mask-wearing, it may require more sustained visual processing. This phenomenon is particularly evident in the case of recognizing fear, given its complexity.

In summary, our study provides valuable insights into the dynamic interplay of factors affecting gaze fixation during emotion recognition in young children. These findings contribute to understanding how facial occlusion contexts, such as mask-wearing, constrain emotion recognition processes during early childhood. Future research in this domain will undoubtedly deepen our understanding of these intricate processes.

Future research may further examine how task relevance interacts with developmental factors in shaping children's emotion processing under conditions of facial occlusion.

### Limitations

This study had several limitations. First, although the sample size exceeded the minimum sample size estimated through an *a priori* power analysis, the study may still have been underpowered to detect very small or higher-order interaction effects. Future studies with larger and more diverse samples would enhance statistical sensitivity and strengthen the generalizability of the findings. Second, the recruitment process, which involved seeking participation from selected kindergartens and daycare centers, introduces the potential for selection bias. Institutions willing to participate might possess unique characteristics that could influence the study's outcomes, and this aspect requires careful consideration.

Finally, the study was conducted during the COVID-19 pandemic, a period characterized by widespread mask-wearing. While the study assesses the impact of masks on emotion recognition, it does not explicitly address whether the observed effects are context-specific or possess broader implications for children's overall development of emotion recognition. Moreover, we did not assess children's momentary affective state, even though recent research suggests that children's own emotional state can bias how they recognize emotions from masked faces (Mastorogianni et al., 2024).

The present study did not assess individual differences in mask exposure or duration of mask-wearing in daily life. Therefore, conclusions regarding developmental adaptation to pandemic-related conditions cannot be drawn from the current data.

## Conclusions

This study provided comprehensive insights into the intricate process of emotion recognition in young children. The findings from both the accuracy assessment of recognizing facial expressions and the analysis of gaze fixation patterns during the recognition process have shed light on the multifaceted nature of this developmental milestone.

Regarding the accuracy of emotion recognition in facial expressions, several critical points have emerged. First, 3- and 5-year-olds exhibited varying levels of accuracy in recognizing emotions. The presence of masks significantly affected their recognition, with unmasked faces yielding higher accuracy. Furthermore, the ability to recognize different emotions varied, with positive emotions such as joy being recognized more accurately than negative emotions, particularly sadness and anger, while fear remained the most difficult emotion overall. These results align with those of prior research, emphasizing the developmental progression in recognizing emotions and the influence of mask-wearing on this ability.

The consistent accuracy in the recognition of joy across age groups suggests early sensitivity to positive emotions, while challenges persist in recognizing negative emotions. The recognition of sadness appears to be a developmental task, progressively improving between the ages of three and five. The findings also indicate that while both age groups could recognize fear to a similar degree, this emotion remains challenging to discern, possibly due to its infrequent occurrence in young children's daily lives.

The analysis of gaze fixation patterns provides converging evidence regarding children's visual strategies during emotion recognition. Although no significant main effect of age emerged, the interaction among mask-wearing, emotion type, and AOIs indicates that gaze allocation is context-sensitive rather than developmentally uniform across this age range. Under unmasked conditions, the eye region served as the primary source of diagnostic information, particularly for emotions such as sadness, anger, and fear. These findings are consistent with prior literature highlighting the functional dominance of the eye region in processing emotionally salient facial cues. Additionally, the influence of mask-wearing on gaze fixation was evident, with participants extending and reducing their gaze on the eyes and mouth, respectively. This behavioral pattern is more plausibly interpreted as a perceptual adjustment to reduced lower-face visibility rather than as evidence of broader socio-developmental change.

## Data Availability

The data analyzed in this study is subject to the following licenses/restrictions: the datasets generated and analyzed during the current study are not publicly available due to ethical restrictions involving research with minors and the presence of potentially sensitive eye-tracking data. Access to the data may be considered upon reasonable request to the corresponding author, subject to approval by the Institutional Review Board. Requests for access to the datasets should be directed to the corresponding author, Chunghee Chung (email: chchung@knu.ac.kr).
