# Correction: Impact of mask-wearing on emotion recognition accuracy and fixation duration in young children

**DOI:** 10.3389/fpsyg.2026.1840146

**Published:** 2026-04-30

**Authors:** Jeongeun Lee, Hyorim Lee, Minsol Kim, Chunghee Chung

**Affiliations:** 1 Busan Gender Equality and Family, Lifelong Education Institute, Busan, Republic of Korea; 2 Department of Home Economics Education, Kyungpook National University, Daegu, Republic of Korea; 3 Center for Beautiful Aging, Kyungpook National University, Daegu, Republic of Korea; 4 School of Child Studies, Kyungpook National University, Daegu, Republic of Korea

**Keywords:** childhood development, COVID-19, emotion cognition, eye movement, facial expressions, masks

In the published version of this article, an asterisk (*) was inadvertently placed next to Hyorim Lee's. In addition, the affiliation for author Jeongeun Lee was incorrect: Busan Gender Equality and Family, Busan, Republic of Korea. The correct affiliation is mentioned below:

Busan Gender Equality and Family, Lifelong Education Institute, Busan, Republic of Korea.

The original version of this article has been updated.

