# Supplementary material for: Impact of mask-wearing on emotion recognition accuracy and fixation duration in young children
Source: Front Psychol. 2026 Mar 24;17:1788919. doi: 10.3389/fpsyg.2026.1788919 (PMC13053317; doi:10.3389/fpsyg.2026.1788919)
Supplement: Supplementary file 1 [file Table_1.docx]

**Supplementary Table S1.**
Bonferroni-adjusted pairwise comparisons for emotion recognition accuracy across age groups, emotion types, and mask conditions. Effect sizes are reported as Cohen’s d

| **Comparison** | **Condition** | **t** | **df** | **p_adj** | **Cohen’s d** |
| --- | --- | --- | --- | --- | --- |
| Joy vs. Sadness | Age 3 | 5.15 | 79 | < .001 | 0.57 |
| Joy vs. Fear | Age 3 | 3.31 | 79 | .039 | 0.37 |
| Joy vs. Fear | Age 5 | 5.38 | 79 | < .001 | 0.60 |
| Sadness vs. Fear | Age 5 | 3.26 | 79 | .046 | 0.36 |
| Anger vs. Fear | Age 5 | 4.51 | 79 | < .001 | 0.50 |
| Sadness (Age 5 vs. Age 3) | Between age | -4.31 | 79 | .001 | 0.48 |
| Anger (Age 5 vs. Age 3) | Between age | -3.30 | 79 | .040 | 0.37 |
| Joy (Unmasked vs. Masked) | Mask effect | 6.91 | 79 | < .001 | 0.77 |
| Anger (Unmasked vs. Masked) | Mask effect | 3.32 | 79 | .038 | 0.37 |
| Sadness (Unmasked vs. Masked) | Mask effect | -1.03 | 79 | .311 | 0.11 |
| Fear (Unmasked vs. Masked) | Mask effect | 2.14 | 79 | .072 | 0.24 |
| Joy vs. Sadness (Unmasked) | Emotion contrast | 7.28 | 79 | < .001 | 0.81 |
| Joy vs. Anger (Unmasked) | Emotion contrast | 3.42 | 79 | .028 | 0.38 |
| Joy vs. Fear (Unmasked) | Emotion contrast | 6.96 | 79 | < .001 | 0.77 |
| Anger vs. Sadness (Unmasked) | Emotion contrast | -4.39 | 79 | < .001 | 0.49 |

Note. p-values are Bonferroni-adjusted for multiple comparisons.

**Supplementary Table S2**

Estimated marginal means (M), standard errors (SE), and 95% confidence intervals for emotion recognition accuracy across mask conditions and emotion types.

| **Emotion** | **Mask Condition** | **M** | **SE** | **95% CI** |
| --- | --- | --- | --- | --- |
| Joy | Unmasked | 0.92 | 0.02 | \|  \| \| --- \|  \| [0.88, 0.95] \| \| --- \| |
| Joy | Masked | 0.74 | 0.03 | [0.69, 0.79] |
| Anger | Unmasked | 0.83 | 0.03 | [0.78, 0.88] |
| Anger | Masked | 0.73 | 0.03 | [0.68, 0.79] |
| Sadness | Unmasked | 0.69 | 0.03 | [0.64, 0.75] |
| Sadness | Masked | 0.72 | 0.03 | [0.66, 0.79] |
| Fear | Unmasked | 0.70 | 0.03 | [0.65, 0.76] |
| Fear | Masked | 0.63 | 0.03 | [0.56, 0.69] |
